# Condition and Honey Productivity of Honeybee Colonies Depending on Type of Supplemental Feed for Overwintering

**DOI:** 10.3390/ani13030323

**Published:** 2023-01-17

**Authors:** Antonín Přidal, Jan Musila, Jiří Svoboda

**Affiliations:** Department of Zoology, Fishery, Hydrobiology and Apidology, Faculty of AgriSciences, Mendel University in Brno, Zemědělská 1, 613 00 Brno, Czech Republic

**Keywords:** beekeeping, honeybee colony, feeding, syrup, inverted sugar, sucrose, growth, strength

## Abstract

**Simple Summary:**

Honeybee colonies are fed by artificial sugar feeds for successful overwintering. There are several kinds of feeds. Traditionally, sucrose from sugar beet or cane is used, with good results. Currently, the inverted sugar syrups containing simple sugars made from starch or sucrose are recommended in spite of being more expensive. The invert syrups are tendentiously considered as a feed which has the potential to improve the condition of a colony before winter, because supposedly the bees are not forced to produce enzymes for the cleaving of sucrose when they are consuming simple sugars. Hence, the objective of this study was to compare the honeybee colonies winterized on stores from invert syrup with those winterized on sucrose as a conventional feed, to find out if the invert syrup has the potential to improve the overwintering of colonies and their following development and production. No beneficial or harmful effects of the invert feed in comparison with the sucrose one were found. We conclude that inverted sugar syrups, with respect to the higher price, are less appropriate for winter supplementation of honeybee colonies in comparison with sucrose feeds.

**Abstract:**

Harvested honey is usually replaced by an alternative sugar to overwinter honeybee colonies. Supplementation of winter stores with beet or cane sucrose is safe for colonies and does not cause winter mortality. Despite this, there are hypotheses that supplementation of inverted sugars has the potential to give better results in overwintering, spring growth, and honey production of the colonies, because bees are consuming already cleaved feed. Therefore, we compared the condition parameters and honey production in 70 colonies at four apiaries overwintered with stores from sucrose or inverted sugars. No statistically significant differences in dependence on the type of the supplemental feed were found. Inverted sugar was more expensive than sucrose for feeding colonies. Economic efficiency, physiological consequences, and other disadvantages of using invert syrups are discussed.

## 1. Introduction

The supplementation of honeybee colonies with sugar feed as a substitute for the harvested honey is one of the standard treatments beekeepers apply to colonies before winter to stock honeybee colonies [1,2]. The conventional source of sugar for substitution of the winter stores were beet or cane sucrose for a long time [1,3]. The longevity of caged bees fed pure sugars (glucose, fructose, sucrose, and maltose in a honey natural ratio) was longer by 16 days than that of bees fed honey [4]. Barker [5] stated that there are no sugars better than pure sucrose, and feeding with syrups from starch is very risky. The quality of syrups from starch at the beginning was poor and contained residues of indigestible starch and/or 5-hydroxymethyl-2-furaldehyd (HMF) which are harmful to bees [6,7]. High-fructose corn syrup (HFCS) shortened the lifespan of workers in cages in comparison to sucrose [8]. The technology to produce the syrup has gradually improved [9] and therefore Severson and Erickson [10] later found that HFCS did not adversely affect the productivity of the honeybee colony. Recent studies have confirmed that colonies wintered on HFCS were weaker in spring (brood production and adult bee population) than colonies wintered on sucrose feed [11]. The reason for these differences in the field experiments could have arisen from the variable composition of HFCS with various sugar residues which are indigestible for the bees, such as fructosyl-fructoses and possibly fructosyl-glucoses [12].

The most important factors for the beekeeper when deciding what kind of feed to use are price, and the health of colonies [5,13]. Therefore, syrup producers now claim in general that supplementation with syrups containing monosaccharides, such as glucose and fructose in an optimal ratio (invert syrup), improves the condition of overwintered colonies [14]; i.e., better colony development in the spring and, consequently, a higher honey production because the processing of monosaccharides physiologically exhausts the bees less than disaccharide (saccharose/sucrose), which has to be enzymatically cleaved. This claim was inspired by the results of Melnichuk [15,16]. He found that processing sucrose syrup in autumn depleted the hypopharyngeal glands of winter workers in proportion to the quantity of inverting enzymes used, reducing the length of life of the bees by up to 25%. Colonies equipped with already capped sucrose stores in combs processed in another colony developed better in the spring than colonies supplemented by feeding with sucrose. However, Jachimowicz [17] proposed that the bees add enzymes in the same amount, regardless of the sugar composition of the feed. This assumption that the bees added invertase to the feed, irrespective of its composition, was later partially confirmed under artificial conditions in the flight room [18].

The first field experiment on supplemental feeding of invert sugars (produced by acid hydrolysis of sucrose) compared with sucrose feeding was carried out in 1982 [19]. This study concluded that feeding with invert syrup provided no benefits but also no risks. Results without any favorable effect of the invert syrup on the overwintered colonies were achieved subsequently [20,21,22]. Severson and Erickson [10] found that colonies supplemented with HFCS had less capped brood surface in spring compared to colonies supplemented with sucrose, similarly to Sammataro and Weiss [11], but had no effect on subsequent honey production. In sum, none of the alternative feeds in the form of various invert syrups had any favorable effects, neither in vitro (cages) nor in vivo (field tests), in spite of the fact that some invert syrups showed higher residual enzymatic activity, which could hypothetically improve the condition of the winter colony. Some risks leading to higher winter mortality may appear if a feed contains a high content of glucose or residues of maltodextrin due to crystallization of the winter stores [23,24]. The serious damage on the epithelial layer in mesenteron was found in bees fed with invert syrup produced by acid hydrolysis [25].

Despite the above knowledge and the higher prices of hydrolyzed syrups, producers recommend them and beekeepers are still interested by them. Hence, the objective of this study was to compare the honeybee colonies winterized on stores from invert syrup produced by enzymatic hydrolysis with those winterized on sucrose as a conventional feed to find out if invert syrup has the potential to improve the overwintering of colonies and their following development and production.

## 2. Materials and Methods

### 2.1. Experimental Design—Apiaries, Colonies, and Hives

Seventy western honeybee colonies (*Apis mellifera carnica* P.) stock Vigor^®^ were tested at four locations in Moravia and Silesia in the Czech Republic (Table 1). The locations were chosen with respect to different environmental factors, because these factors can play a role [26]. The types of used hive are described in Table 1. Individual apiaries were always equipped with only one type of hive. The queen excluder was not used. The colonies were divided into two consistent halves in each apiary according to their condition: the first one as a control group supplied with sucrose syrup, and the other as an experimental group supplied with invert syrup. In both groups, only colonies comparable in terms of their condition and history of their development were included. This means that the number of swarms (colonies established from swarms in this season), splits, and standard stock colonies was the same in both groups at individual apiaries. The condition of colonies were compared according to strength (number of occupied frames) and brood surfaces before feeding period in the subjective mode of BEEBOOK [27]. Similar colonies were equally divided/paired into control and experimental groups. Overwintering of colonies was carried out in hives without honey supers.

### 2.2. Feeds and Feeding

The supplemental feeding of both tested feeds was carried out in late summer (July and August 2014) after the last honey harvest (Table 2). We used a glass hive-top feeder with a perforated plastic cap and a volume of 3.7 L. The sucrose syrup contained 3 parts of crystalline sucrose (from sugar beet) to 2 parts of water. The invert syrup produced by enzymatic hydrolysis of sucrose from sugar beet contained: (a) 28% of water and (b) in dry mass 30% sucrose, 39% fructose, 30% glucose, other sugars <1%, without residues of starch (oligosaccharides and maltose) and HMF 1.8 mg·kg^−1^. The same producer’s batch of the invert syrup and sucrose was used in experiment. 

The same batches of the both invert syrup and sucrose was used in the experiment. Each colony in the both groups was supplied with 18 kg of sugar per colony in total (calculated as the dry mass of feeding solution).

### 2.3. Feed Intake

The honeybee colonies at apiary Brno (separate group of colonies) were chosen to find how fast the colony is able to consume the invert and sucrose syrups and a solution of a mixture of crystalline sucrose and water. Each group consisted of 10 colonies. This mixture was fed through a perforated seepage feeder from which the sucrose crystals were dissolved by the sucking of water from the mixture by bees. A beekeeper does not need to dissolve sucrose to make a syrup, and feeders are filled only by water and the crystalline sucrose in the required ratio. The time consumption was recorded after the second and third refilling of feeders. The process of feed intake of the all three feeds and time of emptying of the feeders were checked 3 times per day (at 7 a.m., 13 p.m, and 19 p.m.) including watching the behavior of bees around the hives indicating eventual robbery attempts [1].

### 2.4. Overwintering

The quantity of dead bees, having fallen with the debris onto the hive bottom during overwintering (winter bee mortality), was evaluated shortly before spring flight and removal of the protective grid against rodents from the hive entrance. It was expressed as a rate of coverage by dead adult workers at the bottom of the hive on a scale from zero to four: 0—no bees, 1—a few individual bees (dozens), 2—the bottom sparsely but regularly covered (about one hundred), 3—the bottom very densely covered in one layer (hundreds), and 4—the bottom densely covered at least partially in layers (near or over one thousand).

The total remainder of winter stores was evaluated after the first spring flight in favorable weather and expressed in kilograms, assuming one dm^2^ of bilaterally sealed store cells represented 0.25 kg of sugar stores. The colony strength was also evaluated after the first spring flight in favorable weather and was expressed as follows: (i) the number of supers occupied by bees, (ii) the position of predominantly occupied super (counted from the hive bottom), (iii) the number of occupied frames, (iv) the comb surface with brood expressed in dm^2^ (brood area), (v) the total number of brood combs, and (vi) the ability of the colony to overwinter, subjectively, by the same person on a scale of 1–10, where 10 was the highest ability/overall condition at the first spring inspection of the colony and zero meant colony death. We have been using this subjective parameter in our breeding program for a long time, and it reflects well the subsequent ability of a bee colony to grow.

### 2.5. Colony Growth and Swarming Tendency

The condition of the colony was evaluated during the blossoming of *Prunus avium* (late spring) from 24 April to 8 May 2015. The following was observed: (i) the number of occupied frames, (ii) the total number of brood combs, (iii) the total brood area (dm^2^), and (iv) the increase of the brood area compared to early spring. The tendency to swarm was evaluated on a 0–4 scale (0—no attempt; 1—queen cups with eggs of one-day-old larvae; 2—fully developed queen cells, 3—capped queen cells, and 4—swarmed out). No anti-swarm treatments were performed.

### 2.6. Total Hemolymph Protein Quantification

Proteins are stored internally in the bees’ bodies [28] primarily in the fat body, hemolymph, and hypopharyngeal glands [29]. To evaluate a potential colony condition depending on the type of winter food, the total hemolymph protein was measured using the Bradford method [30].

The hemolymph was collected in autumn (29 October 2014) and early spring (18 March 2015). Thirty workers from each experimental colony were sampled in plastic vials. The bees were taken from the central part of the cluster/nest. The plastic vials with bees were cooled up to 4 °C and transported to the laboratory for hemolymph collection. Hemolymphs were collected from each sampled worker using a micro-capillary pipette and incision between the 3rd and 4th abdominal tergites in volume 1 µL, to create pooled hemolymph samples with a total volume of 30 µL representing a colony. For quantification of the hemolymph protein of a colony, 1 µL of hemolymph taken from the vortexed pooled sample was mixed with phenylthiourea and phosphate buffer (pH = 7) to prevent hemolymph melanization and to improve the stability of the hemolymph proteins.

Bovine serum albumin (BSA) was used as a standard and to validate the method. Validation of the method using the BSA standard always took place before each determination (the standard was always prepared fresh). Ready-to-use protein reagent Dry Reagent Concentration (BIO-RAD, California, USA) was purchased. This reagent was diluted 1:4 with MilliQ water before analysis (the reagent was always prepared fresh). Determination procedure: 10 µL of the sample was pipetted into a 96 well microtiter plate Nunc Immuno (Fisher Scientific, Pardubice, Czech Republic). Subsequently, 200 µL of diluted protein reagent was added to 10 µL of the sample, followed by incubation for 5 min at room temperature and absorbance measurement at 595 nm using instrument Infinite M200Pro (Tecan, Männedorf, Switzerland). Each sample was analyzed three times.

### 2.7. Honey Yield

The productivity of the colonies was based on the rate of capping of the honey combs, assuming that 1 dm^2^ of capped comb contains 0.25 kg of honey. The error of this method was about 1–3% from the total extracted honey. The honey harvest at apiaries A and D was carried out twice: first at the start of June 2015 and the second time at the end of July 2015, because there was an intensive honey flow also present in May. The honey productivity was evaluated as the amount of honey until early summer, and the total yearly honey production (annual). At the apiaries B and C, spring and early summer honey flows were somewhat weak; therefore, the harvest was carried out only once at the end of July and the honey productivity was only evaluated annually.

### 2.8. Economic Efficiency

The economic efficiency was calculated from the Czech retail prices of the crystalline sucrose sugar from beet (20 CZK/kg) and inverted sugar syrup (27 CZK/kg; Apivital^®^ manufactured in Gyártó for Stech Ltd., Dobruška, Czechia) in 2014. It was calculated for 98% of dry mass of the sucrose sugar and 72% of the invert syrup. Accordingly, in order to supply 1 kg of dry mass per colony it was necessary to feed 1021 g of crystalline sucrose and 1389 g of invert syrup. For sufficient winter supplementation of a colony under the conditions in the Czech Republic, it is usually necessary to feed 18 kg of dry mass of sugar per colony on average. The increased costs for transport and storage of the more voluminous invert syrup, or its potential short storability resulting from its high water-content (risk of spontaneous fermentation), were not included in the calculation.

Some beekeepers [9,31] claim that feeding with invert syrup is easier than sucrose, which usually has to be dissolved before filling the feeders. Therefore, the time necessary to feed the colonies both the invert and sucrose syrup (timeframe) was measured. The feed was always taken from the retail package (25 kg). The timeframe was measured three times at the apiary A by a one person using a stopwatch.

### 2.9. Statistical Analysis

Statistical analysis was performed using SAS software [32] with the null hypothesis that the used feed had no effect on the measured parameters characterizing honey bee colony development and productivity. The values in tables or graphs represent mean ± standard deviations (SD) and brackets represent the size of the samples (n, colonies). Data were tested with the Student’s independent two-sampled and two-tailed test to analyze the effect of the type of feed. The feed intake was analyzed by one-way ANOVA and post hoc analysis was performed using Tukey’s test in case of significant difference of GLM (*p* < 0.05). Data on an ordinal scale (winter bee mortality, ability to overwinter, and swarming tendency) were evaluated with the non-parametric Mann–Whitney test using median and interquartile range in square brackets.

## 3. Results

### 3.1. Feed Intake

**Figure 1:**
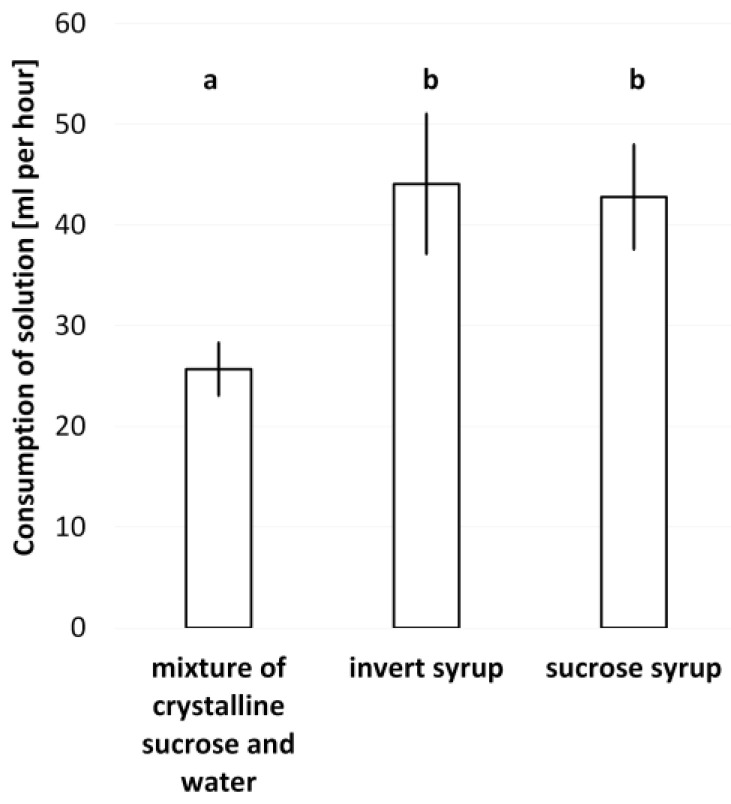
Time needed for intake of a given feed. Means ± SD (bars) and different letters indicate significant differences in consumption speed (Tukey’s test *p* < 0.01).

The rate of feeder emptying (consumption speed) was significantly dependent on the type of feed (Figure 1). The consumption of the mixture was about 40% significantly slower than the liquid forms of the solutions (F = 38.44; df = 2, 27; *p* < 0.001). The sucrose syrup, fully dissolved sucrose, was imbibed by the bees at a very similar rate to the invert feed. No undesirable activity of the bees was observed around the hive entrances during and after filling the feeders and during consumption of the invert syrup or the mixture, unlike the sucrose syrup, which distinctly irritated bees. Therefore, it was not proven that the invert syrup had a greater potential to induce stealing.

### 3.2. Overwintering

The winter bee mortality (Table 3) was 1.2 ± 0.5 in the group fed with sucrose and 1.3 ± 0.6 in the group fed with invert syrup, with a statistically insignificant difference (*p* = 0.626, t-value_68_ = 0.490). The winter mortality did not exceed 150, with the exception of one colony fed by sucrose in Hertice u Opavy, with more than one thousand dead bees (HV7 = 1124). Thus, no negative impact of the tested invert syrup on winter bee worker mortality was observed. The total remainder of winter reserves in early spring (Table 3) was 9.9 ± 4.2 kg in the invert group and 10.1 ± 3.7 kg in the sucrose group again with a statistically insignificant difference (*p* = 0.769, t-value_68_ = 0.295). Similar results were found for brood area and all other spring assessment parameters (Table 3). There were no significant effects from the invert syrup on the winter bee mortality and the total remainder of winter reserves. No winter mortality of a whole colony was recorded.

### 3.3. Colony Growth and Swarming Tendency

There were no statistically significant differences in all five growth parameters of colonies between the invert and sucrose groups (*p* > 0.05) (Table 4). No effect of the feed type on the spring growth of colonies was shown. To present the results according to apiaries, Table 5 was compiled. There were somewhat higher differences between the invert and sucrose group in the brood area and the increase in the brood area. Some were in favor of the sucrose group (apiaries B and C) and others in favor of the invert group (apiaries D and moderately A). In general, none of the differences among groups were statistically significant in assessment parameters or apiaries.

The swarming tendency of the colonies at each apiary was low, probably due to the year 2015 not being a typical swarming season; therefore, no colony showed the tendency to swarm over degree two of the method scale.

### 3.4. Total Hemolymph Protein Quantification

**Figure 2:**
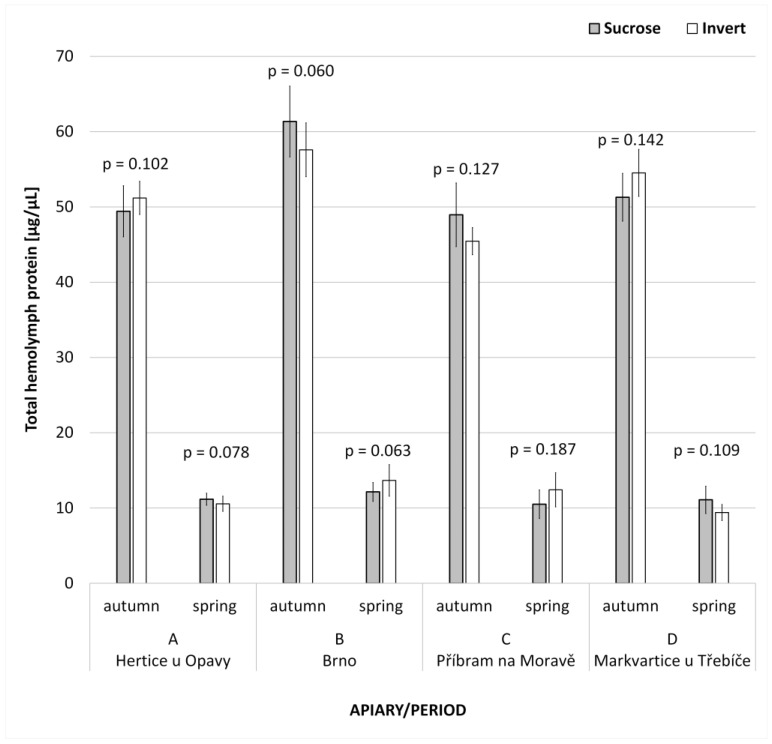
Total worker hemolymph protein (mean ± SD) in dependence of the type of supplemental feed, sorted according to season and location. The differences are, from a physiological point of view, small and statistically insignificant (*p* > 0.05, *t*-test), thus, no effect of the invert syrup was proved.

The total hemolymph protein is summarized in Table 6, including statistical evaluation. Differences in dependence on the type of supplemental feed were not statistically significant for both the autumn and the spring period. The total mean hemolymph protein was significantly higher (*p* < 0.001; t-value_138_ = 57.845) in the autumn (52.9 ± 5.7 µg/µL) in comparison with the spring values (11.4 ± 1.8 µg/µL). The results according to the apiaries (Figure 2) more or less follow the above evaluation. There were not any physiologically substantial nor statistically significant differences.

### 3.5. Honey Yield

**Figure 3:**
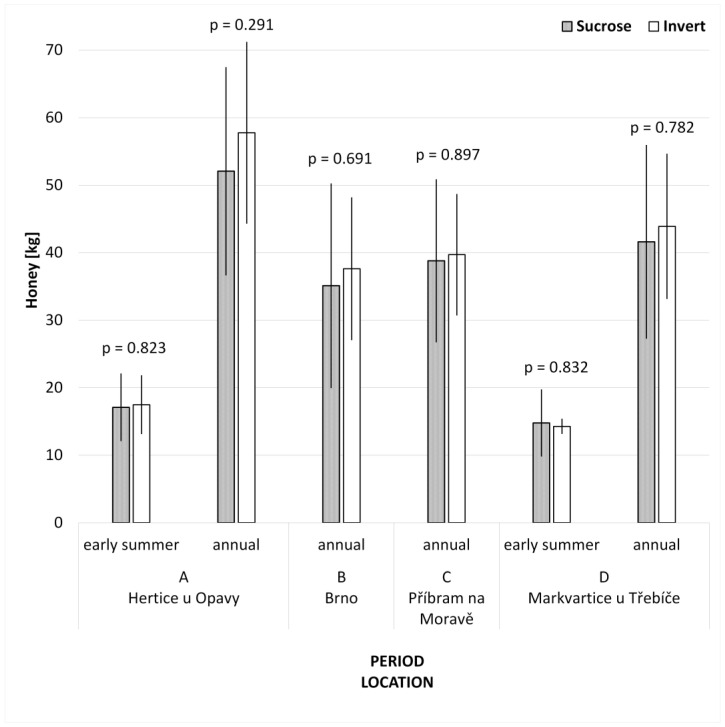
T Honey yield (mean ± SD) independent of the type of supplemental feed, sorted according to season and location. The differences are, from a production point of view, small and statistically insignificant (*p* > 0.05, *t*-test); i.e., no effect of the invert syrup was proved.

The annual honey yield in the sucrose group was 43.8 ± 16.0 kg on average and in the invert group 47.4 ± 14.6 kg. This difference of 3.6 kg is not statistically significant (*p* = 0.328, t-value_68_ = 0.986). The honey production at individual apiaries and with respect to the early honey flow if one was recorded (there was almost no early summer honey flow at apiaries B and C) is depicted in Figure 3. In particular, differences between groups in the mean of the early-summer honey yield were negligible, and did not exceed 0.5 kg. The higher difference in the annual honey production was found at apiary (A) Hertice u Opavy, i.e., +5.7 kg of honey in the invert group. This difference also is not statistically significant (*p* = 0.291, t-value_28_ = 1.075).

### 3.6. Economic Efficiency

Feeding invert sugar was more expensive (by 184%, i.e., by 308 CZK per colony) than feeding sucrose. The costs were still about 157% higher (i.e., 208 CZK) in spite of the lower price of the high-volume packet (1400 kg; 23 CZK/kg) used for calculation. The time spent feeding the invert syrup was about 3.9% longer than feeding sucrose under the experimental conditions.

## 4. Discussion

The consumption rates of the invert syrup and sucrose syrup were very similar, and the difference was insignificant. The results by Free and Spencer-Booth [33] could explain the somewhat faster consumption of the invert syrup, where the bees willingly accepted a more concentrated sugar solution. The invert syrup feeding did not excite bees to robbing. The sucrose solution stimulated bees to more activity around the hive entrance, unlike the mixture, especially when the sucrose solution was fed at noon during sunny weather. It seems that the invert syrup or the mixture is not as attractive or exciting for bees as the sucrose syrup, which is consistent with the results by Barker and Lehner [34].

As assumed by Melnichuk [15,16], the strength of the colonies overwintered on the invert syrup should be potentially better when this feed does not need to be cleaved with their enzymes by bees, unlike sucrose. Inverted sugar feed has a lower potential to exhaust workers within its supplementation. However, the results presented here show that the strength of colonies supplemented with the invert syrup did not differ statistically from those who were sucrose-fed, which is consistent with former experiment by Ceksteryte and Racys [21]. The subsequent development of colonies in late spring could be influenced by other factors differing among the apiaries (mainly depending on local nutritional sources).

There is a general postulate among beekeepers [14] that colonies supplemented with invert syrup for winter periods should grow stronger in the spring, because their workers are not forced to produce enzymes needed for the cleavage of disaccharides. In theory, such workers should be less physiologically exhausted. However, no significant differences were found in the spring colony growth when comparing colonies from the control and experimental group, thus, the potential prevention of the physiological exhaustion of the colonies (supplemented with cleaved sugars on the spring growth) was not confirmed. The presumptive effect of the invert syrup was not supported, even by small or statistically insignificant differences, in: (a) the mean total hemolymph protein in workers or (b) the swarming tendency. Similarly, Guler et al. [35] recommended not using industrial sugars, except sucrose, for winter feeding of colonies, because all tested syrups had negative effects on overwintering ability when comparing to sucrose-fed groups. They stated that for good colony management and to produce a strong colony, sugar types and syrup levels, as well as colony nutrition, are of great importance.

Differences in the honey production between the control and the experimental colonies were not statistically significant; moreover, the honey production in the early summer (first and early honey flow) was almost identical in the both groups. This is consistent with results from similar experiments [11,19]. The values from the early honey flow are more reliable for an assessment of the colony condition after winter than from the summer or late summer production. The later honey production can be affected also by subsequent factors based on the character of the summer honey flow when a colony builds its fitness for the next season. The honey production from the early honey flow is potentially affected by the previous winter colony condition more than the later summer production.

The claim that supplementation with an invert sugar can prevent exhaustion in the winter worker bees [14] was probably tendentiously inspired by the results of Melnichuk [15,16]. So far, there is no direct evidence to support this hypothesis. Jachimowicz [17] presupposed and Bacílek et al. [18] subsequently proved that the bees add a similar amount of enzyme to the feed irrespective of its composition. The situation would probably be different if a colony received pre-processed stores without feeding, as proposed by Melnichuk [16]. Skubida [36] found that the later supplemental feeding of colonies in autumn reduced colony development in spring, corresponding with Melnichuk [15,16]. He documented lower bee invertase activity and shorter lifespans in bees (physiological exhaustion of bees) forced to consume sucrose syrup in comparison with bees from an unfed control group. The invertase activity was measured after feeding different quantities of the sucrose syrup, and was expressed as the amount of inverted sucrose by enzyme from a bee head. Wegener et al. [37] found that invertase (alpha-glucosidase) activity in hive bees from experimental colonies fed with sucrose was relatively high. Nevertheless, it does not directly conflict with the results by Melnichuk [15,16], because it is possible that the higher enzyme activity is typical for the initial stage of the exhaustion process during feeding, which can result in the final exhaustion of the enzyme at the end of the processing. Even the present study did not try directly to prove that supplemental feeding mostly with monosaccharides prevents exhaustion of the winter bees; however, concurrently, no improving effect on the following colony condition was observed. Winter supplementation should be completed early [36] enough that the winter bees do not have to prepare or process the food, i.e., that the bees of the summer generation prepare the winter supplies. The winter supplementation in this study was carried out on time, by best practice. Hence, the effect of “exhausted” bees may not have been visible, because these were not the tested bees that prepared the winter stores. The situation could be different in the case of delayed feeding by wrong practice.

Cost is the primary consideration in feedstuffs [5]. High-fructose syrups produced by the enzymatic fermentation of corn starch have become available at a lower cost than sucrose [38]. However, the overwintering colony needs sugar stores without indigestible components such as oligosaccharides or maltose [20,23] or the toxic substance HMF [6]. Thus, high qualitative demands on bee feeds require an excellent quality of substrates which are closer to crystalline sucrose and processing by enzymatic rather than acid-hydrolyzation. These requirements for well-inverted sugars increase the production costs; therefore, feeding with invert syrups is not usually cheaper [39] in comparison with feeding with sucrose (the same in this study). Neither the declaration of producers nor some beekeepers regarding less laborious feeding with primarily liquid feed, such as invert syrups [9], was confirmed under the conditions of the presented experiment. In addition, there are risks with the high content of HMF, starch, or glucose surplus in poor-quality hydrolysates, which can cause an increase in winter colony mortality [23]. These complications are prevented in the case of feeding with crystalline sucrose [5] which is good for storage, due to both the low volume and the long durability. Starch syrups change within storage, mainly in regards to the increasing of HMF content [40].

The technology of commercial feed syrups for bees, namely during acid hydrolysis [41], can cause the formation of HMF. Results by Ceksteryte and Racys [21] suggested that the bee’s body is able to metabolize the HMF to some extent. However, metabolism of HMF in the digestive tract in the honeybee has not been studied, unlike in mammals [42]. The metabolite 5-sulfoxymethyfurfural is genotoxic [43] and may act as both an initiator and a promoter of colon cancer [44]. Indeed, it is well known that HMF is seriously toxic to honeybees [6,41,45]. An HMF content of 48 mg·kg^−1^ in sugar syrup from maize was harmless for wintering honey bees [21]. Moreover, in comparison with honey, winter nourishment via sugar substitutes can negatively affect bee immunity [46] due to the absence of some biologically active constituents (e. g. p-coumaric acid). Therefore, feed fortification, as in the case of the commercial substitute Pchelit [21], can positively influence spring colony development. There are serious risks when poor or impure sugar substitutes [12] are used for winter supplementation, because a honeybee colony is potentially more susceptible to stress during winter and early spring [47]. It has to be noted that caged bees lived longer on a mixture of pure sugars, in comparison with bees on honey only [4].

The natural enzymatic attributes of honey used for evaluation of its authenticity can be influenced by supplemental feeding [48]. There are two ways to achieve this: (a) decreasing the activity of original honey enzymes such as diastase [49] or invertase [50] by diluting them with the feed, particularly if the winter stores remain in abundance in the nest until following honey harvest [36], or (b) adding foreign/exogenous enzyme(s) that was/were used within the production of an invert syrup [21] (so called residual enzymatic activity in a fed syrup). Therefore, invert syrups can also be produced with specific enzymes, such as beta-fructofuranosidase [51] or exogenous amylases [52,53], and their residues in these syrups can be transferred to the honey afterwards [51]. The presence of such foreign enzymes in honey, as a result of the supplemental feeding of colonies, can be successfully used to detect adulterated honey [54]. Accordingly, there is a risk of unintentional adulteration of the honey due to the colony being fed on enzymatically hydrolyzed syrups. This risk also concerns other beekeepers, because bees are able to bring food from neighboring colonies fed on a syrup. Such transportation among neighboring colonies, as a result of drifting [55] or robbing, is also well known in the case of spreading of bacterial spores of *Paenibacillus larvae* [56] or antibiotics [57]. Some enzymes are highly stable and their long-term activity or high-temperature stability is well known [58].

It can be concluded that low economic efficiency and the discussed uncertain quality, worse storability, and a potential honey contamination with the markers indicating honey adulteration make invert syrups a risky feed.

## 5. Conclusions

It can be concluded that supplemental feeding with inverted sugars, compared with sucrose, has a low potential to improve the colony condition in spring and to increase honey productivity. Further research in the field of the winter nutrition of colonies focused on a potential honey contamination with markers indicating honey adulteration is needed.

## Figures and Tables

**Table 1:** Characterization of apiaries: municipality, GPS location, number of colonies at individual apiaries, and description of used hives (number and dimensions of used frames and supers).

Location & Altitude (above s. l.) GPS	Number of Colonies	Hives Frames and Supers
(A) Hertice u Opavy (325 m) N49° 54′ 22″ E17° 47′ 54″	30	11 pcs (39 × 24 cm) in brood and honey chamber (3–4 pcs without thermal insulated supers)
(B) Brno (234 m) N49° 12′ 38″ E16° 36′ 51″	20	10 pcs (37 × 30 cm) in brood chamber (2 pcs thermal insulated supers) 10 pcs (37 × 17 cm) in honey chamber (2–3 pcs thermal insulated low supers)
(C) Příbram na Moravě (432 m) N49° 11′ 25″ E16° 17′ 38″	10	11 pcs (39 × 27.5 cm) in brood chamber (2 pcs thermal insulated supers) 11 pcs (39 × 17 cm) in honey chamber (2–3 pcs thermal insulated low supers)
(D) Markvartice u Třebíče (565 m) N49° 11′ 58″ E15° 45′ 58″	10	Langstroth ¾, 10 pcs (448 × 185 mm) in 5–6 supers without thermal insulation

**Table 2:** Period of winter stores supplementation at individual apiaries. Dates represent intervals during which the tested feeds were fed.

Apiary	Period
A	29. VII.–7. IX. 2014
B	25. VII.–8. VIII. 2014
C	12. VII.–26. VII. 2014
D	13. VII.–27. VII. 2014

**Table 3:** Condition of just overwintered colonies in early spring. Differences were statistically tested (df = 68) by *t*-test in case of data on a ratio scale and Mann–Whitney test for data on an ordinal scale. No difference was statistically significant in dependence of the type of feed.

Parameters (Units)	Group—Type of Feed (n)		*t*-Test	
	Sucrose (35)	Invert Syrup (35)	*p*-Value	t-Value
Rest of winter reserves (kg)	10.1 ± 3.7	9.9 ± 4.2	0.769	0.295
Brood area (dm^2^)	8.8 ± 5.3	9.6 ± 4.4	0.489	0.696
Brood combs (pcs)	2.3 ± 0.9	2.6 ± 0.7	0.138	1.501
Position of predominantly occupied super *	1.8 ± 0.4	1.6 ± 0.5	0.107	1.633
Supers occupied by bees (pcs)	1.8 ± 0.4	1.8 ± 0.4	0.564	0.580
Occupied frames (pcs)	6.0 ± 1.7	6.6 ± 1.8	0.147	1.467
			**Mann-Whitney test**	
			***p*-Value**	**U-Value**
Winter bee mortality (scale 0–4)	1.0 [0.5]	1.0 [1.0]	0.845	580.0
Ability to overwinter (scale 1–10) **	7.0 [2.0]	7.0 [3.0]	0.344	532.5

* counted from the hive bottom; ** degree “10” means the highest subjectively described the colony condition for an ability to overwinter; n—number of colonies.

**Table 4:** Colony growth in the late spring within blooming of cherry-trees characterized by five monitored parameters describing the amount of adults or brood and tendency to swarm (on an ordinal scale). Differences were statistically tested (df = 68) by *t*-test in case of data on a ratio scale and Mann–Whitney test for data on an ordinal scale. No difference was statistically significant in dependence of the type of feed.

Parameters (Units)	Group—Type of Feed (n)		*t*-Test	
	Sucrose (35)	Invert (35)	*p*-Value	t-Value
Occupied frames (pcs)	16.8 ± 6.2	17.7 ± 7.0	0.604	0.521
Brood combs (pcs)	7.3 ± 3.2	7.6 ± 3.0	0.692	0.398
Brood area (dm^2^)	48.2 ± 19.2	52.5 ± 25.2	0.442	0.773
Increase in brood area (dm^2^)	39.5 ± 17.4	42.9 ± 18.2	0.451	0.758
			**Mann-Whitney test**	
			***p*-Value**	**U-value**
Swarming (degree)	0.0 [0.5]	0.0 [0.5]	0.943	197.5

n—number of colonies.

**Table 5:** Colony growth in the late spring during blooming of cherry-trees and sorted according to apiary A–D and type of feed. Differences among groups were tested as in Table 3 and Table 4 and were not statistically significant at any apiary.

Parameter (Units)	Type of Feed	Apiary (n_1_ + n_2_)			
		A (15 + 15)	B (10 + 10)	C (5 + 5)	D (5 + 5)
Occupied frames (pcs)	sucrose	16.8 ± 1.8	13.4 ± 5.8	15.3 ± 3.2	26.8 ± 10.5
	invert	17.5 ± 1.5	13.1 ± 7.2	13.0 ± 5.7	29.2 ± 5.5
	*p*-value	0.281	0.922	0.526	0.693
	t-value	1.099	0.100	0.663	0.409
Brood combs (pcs)	sucrose	7.3 ± 2.0	5.9 ± 2.6	7.0 ± 2.7	11.0 ± 5.9
	invert	7.5 ± 1.8	5.8 ± 2.9	6.5 ± 2.6	13.0 ± 2.4
	*p*-value	0.709	0.911	0.801	0.560
	t-value	0.378	0.114	0.261	0.608
Brood area (dm^2^)	sucrose	45.2 ± 10.5	40.3 ± 21.3	50.2 ± 14.2	77.4 ± 20.9
	invert	48.8 ± 7.8	30.6 ± 20.9	42.6 ± 23.7	97.8 ± 17.0
	*p*--value	0.289	0.675	0.607	0.167
	t-value	1.081	0.426	0.536	1.520
Increase in brood area (dm^2^)	sucrose	34.4 ± 9.5	35.5 ± 19.7	40.2 ± 13.9	66.3 ± 16.3
	invert	37.1 ± 6.7	38.6 ± 13.6	33.6 ± 18.6	80.5 ± 4.6
	*p*-value	0.390	0.716	0.591	0.179
	t-value	0.874	0.370	0.560	1.473
Swarming (degree)	sucrose	1.0 [1.0]	0.0 [0.0]	0.0 [0.0]	0.0 [0.0]
	invert	0.0 [1.0]	0.0 [0.0]	0.0 [0.0]	0.0 [0.0]
	*p*-value	0.736	0.502	0.551	1.000
	U-value	105.0	8.0	20.0	12.0

n—number of colonies at an apiary in sucrose (n_1_) and invert group (n_2_).

**Table 6:** Total hemolymph protein in autumnal and early spring honeybee workers. Differences were statistically evaluated by *t*-test (df = 68) and t- and *p*-values are presented in the last two columns. No difference was statistically significant in dependence of the used feed.

Total Hemolymph Protein (µg/µL)	Group—Type of Feed (n)		*t*-Test	
	Sucrose (35)	Invert (35)	*p*-Value	t-Value
Autumn	53.0 ± 6.5	52.7 ± 4.8	0.797	0.258
Spring	11.3 ± 1.3	11.6 ± 2.2	0.630	0.483

n—number of colonies.

## Data Availability

Not applicable.
